# A Comprehensive Analysis of Myocarditis in Formerly Healthy Individuals Following SARS-CoV-2 Vaccination (COVID-19 Immunization)

**DOI:** 10.7759/cureus.26851

**Published:** 2022-07-14

**Authors:** Kamal Sharma, Smeet Patel, Zeel Patel, Kalpen B Patel, Jinish S Doshi, Darshini B Shah, Priyank Chokshi, Ansh Parbatani, Chandan Sharma, Akanksha Patel, Ashwati Konat

**Affiliations:** 1 Cardiology, Shah Alloys Limited (SAL) Hospital, Ahmedabad, IND; 2 Graduate Medical Education, Smt. Nathiba Hargovandas Lakhmichand (NHL) Municipal Medical College, Ahmedabad, IND; 3 Graduate Medical Education, Ahmedabad Municipal Corporation Medical Education Trust (AMC MET) Medical College, Ahmedabad, IND; 4 Internal Medicine, Pandit Deendayal Upadhyay Medical College, Rajkot, IND; 5 Internal Medicine, Ahmedabad Municipal Corporation Medical Education Trust (AMC MET) Medical College, Ahmedabad, IND; 6 Medicine, Gujarat Cancer Society (GCS) Medical College, Hospital and Research Centre, Ahmedabad, IND; 7 Internal Medicine, Gujarat Adani Institute of Medical Sciences, Bhuj, IND; 8 Medicine, Government Medical College, Surat, Surat, IND; 9 Internal Medicine, Pramukh Swami Medical College, Anand, IND; 10 Department of Zoology, Biomedical Technology and Human Genetics, Gujarat University, Ahmedabad, IND

**Keywords:** vaccination, sars-cov-2, healthy individuals, myocarditis, covid-19

## Abstract

Due to the rapid development of the coronavirus disease 2019 (COVID-19) pandemic, the Food and Drug Administration (FDA) expedited the authorization of immunizations to counteract life-threatening COVID-19 effects. COVID-19 immunization was seen as an essential component of surviving endemically with COVID-19. Although there were no major adverse event reports that mandated an early authorization of the mass vaccination approval in initial studies, a few significant adverse events were reported after real-world usage. The most prevalent adverse events are regional reactions, such as discomfort at the injection site. Anaphylactic shock and acute responses were quite infrequent. Current evidence strongly convince the community that the advantages of immunization outweigh the risks.

The review investigates the potential adverse reaction in the form of myocarditis caused by the COVID-19 vaccine. Age, sexuality, vaccination type, clinical manifestations, and diagnostic modalities were among the confounding factors associated with vaccine-induced myocarditis. This picture depicts COVID-19 immunization-induced myocarditis and the treatment options available to practitioners. Further evaluation is needed to establish the underlying cause of this association. We compiled the most recent data on SARS-CoV-2 vaccine-induced myocarditis after reviewing available research. Information sources including PubMed and Google Scholar were evaluated retrospectively.

## Introduction and background

Myocarditis has been documented in a small percentage of COVID-19 vaccine recipients worldwide [1]. While the exact etiology of cardiac inflammation is unknown, monitoring data suggests that it is caused by an immune-mediated or hypersensitive reaction. Following COVID-19 immunization, extra safety surveillance is being done to find the true perpetrator [1].

Considering the effectiveness and safety of vaccines including tozinameran and elasomeran, questions about vaccination-induced myocarditis have been acknowledged [1,2]. Following early reports from Israel, the European Medicines Agency (EMA) and the US Food and Drug Administration (FDA) issued a warning concerning the potential of pericarditis and/or myocarditis following immunization against COVID-19 [2]. Commonly, one of the major contributing factors of cardiogenic shock in younger generations is fulminant myocarditis, an uncommon, severe form of cardiac muscle inflammation [3,4].

The US Centers for Disease Control and Prevention (CDC) produced data in August 2021 indicating an increased incidence of immunization-induced myocarditis in relatively younger men. However, no age group difference was evident [5]. The male gender has been identified as a risk factor for mortality in SARS-CoV-2 coronavirus sufferers [6]. Gender differences in immunological responses could influence the outcome of virus-related cardiac disease [7,8].

Oster et al. discovered in a descriptive study that the crude reporting rates of 1,626 cases of myocarditis within seven days of immunization exceeded the expected rates across multiple age and gender strata [9]. In this study, we take a glance at the progression of SARS-CoV-2 vaccine-induced myocarditis, its probable causes, and recovery rates.

## Review

Methods

We investigated case reports and case series that showed myocarditis post-COVID-19 immunization, regardless of the type of vaccine, and we ruled out certain other COVID-19 immunization-related adverse health events that were published online. Out of 40 published articles, we found 26 articles that fit our criteria, with a total of 104 occurrences of myocarditis following COVID-19 vaccination (Table 1) [10-35].

**Table 1:** Brief summary of case reports and case series used as references in this study

#	Researchers	Summary
1	Singh et al. [10]	The researchers focused on the case of a male patient who developed chest discomfort, including a dull aching and a squeezing or burning sensation, after receiving his second dose of the COVID-19 vaccine; the patient was diagnosed with myocarditis. According to the researchers, the benefits of vaccination outweigh the risks because COVID-19 infection causes morbidity and mortality, while recorded cases of myocarditis are mild and infrequent, and most patients survive.
2	Hasnie et al. [11]	The researchers described a case of COVID-19 immunization-related myocarditis in a young male who has been previously exposed to COVID-19 but was otherwise asymptomatic. The case report describes an unusual yet serious adverse occurrence that clinicians should be aware of.
3	Das et al. [12]	COVID-19 vaccination-induced acute myopericarditis was found to be significantly correlated with arrhythmia in this investigation according to the researchers.
4	Rosner et al. [13]	The clinical characteristics of immunization-related myocarditis-like illness appear to be encouraging, with all patients obtaining symptom remission. Given the high risk of COVID-19 infection, especially in children, vaccination remains a very appealing risk-benefit option.
5	Larson et al. [14]	The researchers in this study investigated the usage of corticosteroids in severe clinical myocarditis due to the obvious potential immune-mediated postvaccination mechanism. Corticosteroids, on the other hand, may reduce the specific immunological response elicited by the vaccine against SARS-CoV-2.
6	Vidula et al. [15]	The first case series of patients who developed myocarditis, stress cardiomyopathy, or pericarditis after ingesting the mRNA-based COVID-19 vaccinations has been published. They hoped that reporting these issues will allow for more monitoring and inquiry.
7	Kim et al. [16]	According to the researchers, rapid screening, including cardiac markers, electrocardiography, or imaging, must be required for quick diagnosis in individuals with related symptoms within a week of receiving COVID-19 vaccination.
8	King et al. [17]	A few days after receiving the COVID-19 mRNA vaccine, four people developed myocarditis, which was characterized by chest pain, increased troponin I and C-reactive protein, and negative viral serologies.
9	Abbate et al. [18]	The study describes two occurrences of fulminant myocarditis following COVID-19 immunization, which was linked with systemic hyperinflammatory syndrome and refractory shock requiring extracorporeal membrane oxygenation support.
10	Dickey et al. [19]	In this investigation, the researchers discovered that in six cases with clinical presentation, cardiac magnetic resonance (CMR) data, and temporal correlation strongly suggest vaccine-associated myocarditis.
11	Williams et al. [20]	According to the researchers, patients who showed chest pain after being immunized against COVID-19 with mRNA vaccines appeared to have myocarditis.
12	Bautista García et al. [21]	This report describes a patient with a known case of asthma, autoimmune hypothyroidism, and chronic atrophic gastritis, with the hypothesis that the immunization against COVID-19 was the cause of acute myocarditis.
13	Ramírez-García et al. [22]	The study presented two cases of pericarditis caused by anti-SARS-CoV-2 immunization.
14	Tano et al. [23]	According to the researchers, doctors should look for myocarditis in patients who have a major complaint of chest pain after receiving the COVID-19 vaccine and be aware of the clinical ramifications and the necessity to document this potentially harmful event.
15	Isaak et al. [24]	The cardiac MRI features of COVID-19 immunization-induced hypersensitivity myocarditis are analogous to those of other virus-induced myocarditis, according to this study.
16	Deb et al. [25]	In an elderly male with acute congestive heart failure, the researchers presented a case of acute myocardial damage caused by a sudden immunological reaction following the second dose of COVID-19 vaccination. His health symptoms improved over the course of three days.
17	McLean et al. [26]	The study uses the case of an otherwise healthy adolescent who developed myopericarditis after receiving two doses of COVID-19 vaccination with no other known causes. After 36 hours of sharp/stabbing chest pain following immunization, the patient attended to the ER.
18	Muthukumar et al. [27]	According to this study, a rare hypothetical vaccine-related unexpected outcome does not impact the advantageous risk/benefit ratio of COVID-19 immunization, including those in individuals with underlying cardiac disease.
19	Habib et al. [28]	A 37-year-old male was transported to the hospital three days after receiving the second dosage of the COVID-19 vaccine, complaining of acute chest pain. The pain was crushing and at the back of the neck. Body aches, fever, shaking, and a headache preceded it.
20	Mansour et al. [29]	In this paper, the researchers reported two cases of myocarditis in previously healthy people who received the mRNA-COVID-19 immunization. Both patients experienced severe chest pain, ECG irregularities, and raised serum troponin levels within two days after obtaining their second dose.
21	Watkins et al. [30]	A healthy male went to the hospital with the primary complaint of midsternal chest discomfort. As a result of the pain, the patient also experienced slight shortness of breath. The patient had had his second dose of the immunization two days before the onset of the chest problems.
22	Minocha et al. [31]	The researchers discussed the case of an adolescent who had acute myocarditis four months after experiencing coronavirus-negative acute myocarditis and 48 hours after receiving his second dose of the COVID-19 vaccine.
23	Albert et al. [32]	COVID-19 infection was connected to a substantially higher risk of cardiac involvement than COVID-19 vaccination, according to the researchers.
24	D’Angelo et al. [33]	The researchers described a 30-year-old male who developed dyspnea and retrosternal pain following receiving the SARS-CoV-2 vaccine, which is an uncommon side effect of numerous COVID-19 immunizations. Using cardiac magnetic resonance imaging and laboratory data, the definitive diagnosis of myopericarditis was made.
25	Nassar et al. [34]	The findings of this possible situation established myocarditis as a possible side effect of COVID-19 vaccinations. Early diagnosis is critical for minimizing COVID-19 vaccine-related adverse events and improving treatment options for patients suspected of having myocarditis.
26	Marshall et al. [35]	All instances of myocarditis that occurred post-COVID-19 mRNA immunization should indeed be investigated for acute COVID-19 infection, as well as a comprehensive workup to rule out other infectious and noninfectious causes.

Search Strategies

Using academic sites such as Google Scholar and PubMed, a comprehensive search of COVID-19 immunization-induced myocarditis published studies was conducted. The search approach employed terms such as “Covid-19,” “Myocarditis,” “healthy individuals,” “SARS-CoV-2,” and “Vaccination” to discover targeted reporting items. Boolean operators were used to group the search results (“OR” and “AND”).

Process of Data Collection and Data Items

We retrieved variables such as age, sexual identity, and other covariates, namely, immunization type, clinical manifestations, how long has it been since they were recognized with myocarditis, consequences, laboratory findings, diagnosis strategies, and results, and we independently reviewed all of these variables using standardized data harvesting forms on an Excel sheet (Microsoft Corporation, Redmond, WA, USA).

Result

**Figure 1:**
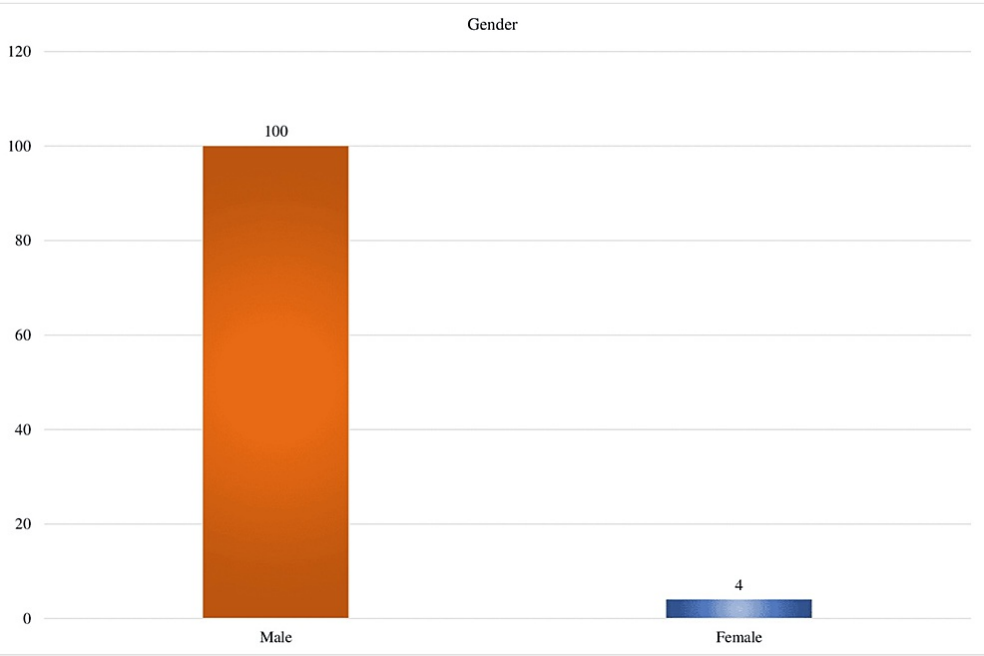
Male versus female demographics affected

The average age of the patients in this study of 104 individuals with COVID-19 vaccine-associated myocarditis was 21 years (interquartile range (IQR): 17-29). Only four of the 104 documented incidents included females, while 100 featured males (Figure 1).

**Figure 2:**
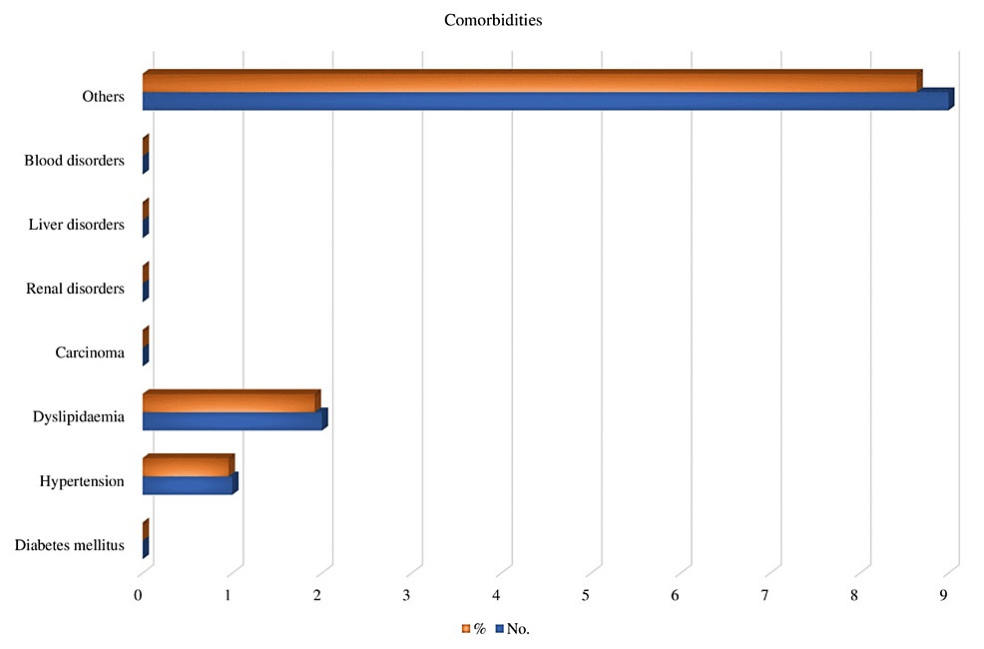
Comorbidities affiliated with the patients and their types

In our research, we discovered that one patient (age: 52 years) had hypertension, two patients had dyslipidemia, nine patients had various comorbidities, and three patients had an addiction (Figure 2).

**Figure 3:**
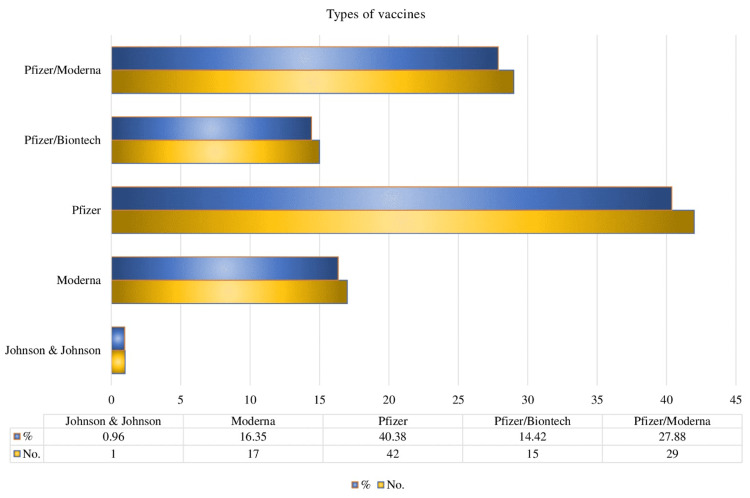
Myocarditis incidence with several types of vaccines against COVID-19

Our reference studies found myocarditis after vaccination with various types of COVID-19 disease vaccines (Figure 3).

**Figure 4:**
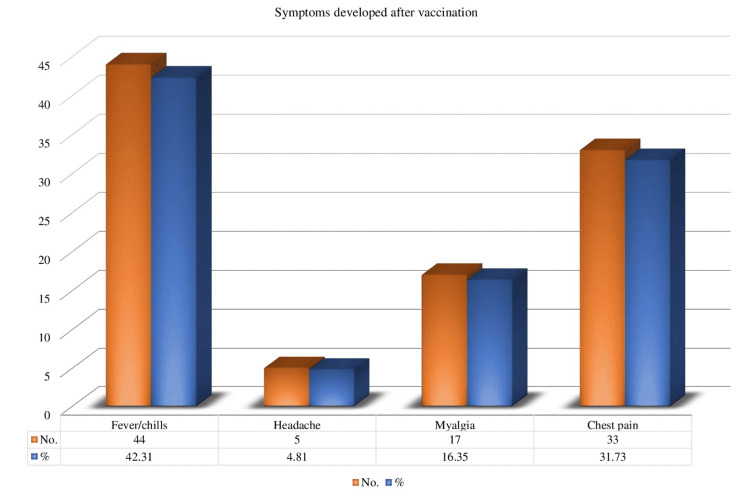
Symptoms that appeared after COVID-19 immunization

Symptoms such as fever, chills, headache, myalgia, and chest pain were observed following vaccination in our study. Out of 104 (89.42%) patients, 93 had received both doses (Figure 4).

In our analysis, we discovered that 58 (55.77%) of 104 cases had troponin peak with a median of 11.40 ng/dL (IQR: 2.72-28.5 ng/dL). In our investigation, we determined that 64 (61.54%) out of 104 patients had a CRP level of 7.60 mg/dL or higher (IQR: 3.96-14 mg/dL).

**Figure 5:**
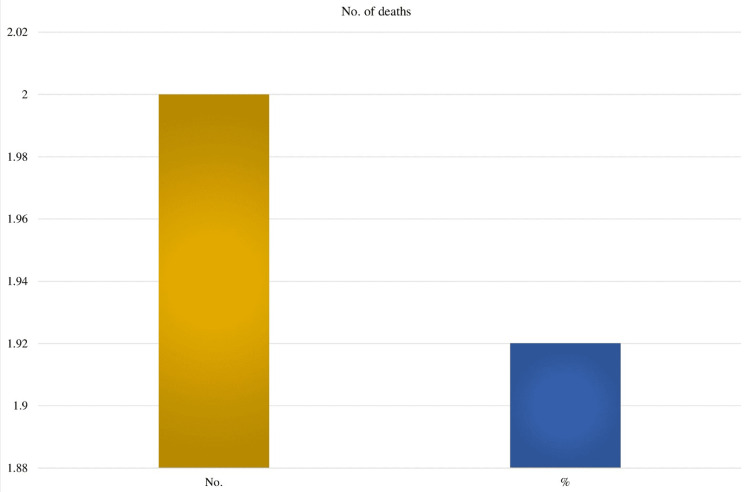
Total deaths reported following immunization against COVID-19

In our review, we reported two deaths (one from refractory cardiogenic shock and the other from renal failure) out of 104 total cases, which accounted for 1.92% of the reported cases (Figure 5).

Discussion

COVID-19 has infected millions of individuals worldwide since it was declared a pandemic by the World Health Organization (WHO). There had been 519,729,804 documented cases of COVID-19 sickness to the WHO as of May 17, 2022, with 6,268,281 deaths and a total of 11,660,363,722 vaccine doses distributed [36].

Myocarditis is a cardiac inflammatory illness caused primarily by viruses and also by protozoa, bacteria, and fungi (such as *Borrelia *spp.). It can be caused by a number of potentially dangerous medicines and therapies (such as immunological checkpoint inhibitors). Other causes involved systemic immunological-mediated illnesses [37,38].

The most common viruses associated with inflammatory cardiomyopathy include adenoviruses and enteroviruses, as well as viscerotropic viruses such as parvovirus B19 and lymphotropic viruses such as Epstein-Barr virus and human cytomegalovirus. Viruses such as HIV, hepatitis C virus (HCV), influenza A/B viruses, and Coronaviridae viruses have been demonstrated to cause myocarditis indirectly through cytokine-induced cardiotoxicity or an autoimmune response against heart components [39-41].

A cytokine cascade induced by an unbalanced interplay of T helper 1 cells (TH1 cells) and T helper 2 cells (TH2 cells) in COVID-19 patients causes heart injury [42,43]. SARS-CoV-2 infection induces respiratory failure and hypoxia, both of which can lead to heart damage [44].

The pathophysiology of vaccine-related myocarditis is assumed to be the immune system misinterpreting the vaccine’s mRNA for an antigen, culminating in pro-inflammatory responses and immunological consequences in the heart. Antibodies produced against SARS-CoV-2 spike glycoproteins might interact with human protein sequences that are physically similar, such as cardiac myosin heavy chains [45].

In clinical and experimental studies, male preponderance in myocarditis cases has already been observed. One possible explanation is differences in sex hormones [46]. By inhibiting anti-inflammatory cells, testosterone is thought to have a role in triggering a Th1 immune response; estrogen suppresses pro-inflammatory T cells, lowering cell-mediated immune responses and therefore the risk of autoreactivity [46].

The presence of pro-inflammatory cytokines including IL-6, IL-8, and tumor necrosis factor-alpha in critically ill SARS-CoV-2 patients implies that complementary system development may influence COVID-19 clinical outcome [47,48].

COVID-19-related cardiac dysfunction or myocarditis, on the other hand, is thought to be 100 times more common than COVID-19 mRNA vaccine-related myocarditis (1,000-1,400 per 100,000 COVID-19-positive patients) [49]. Furthermore, COVID-19 is linked with an increased risk of cardiovascular problems than immunization-related myocarditis, which presents equally mildly and has a favorable prognosis [50].

The most often documented local side effects of the COVID-19 immunization were pain at the insertion site, edema, and warmth. Systemic manifestations included fever, myalgia, fatigue, and headaches. Some trials revealed laboratory anomalies such as decreased hemoglobin, enhanced bilirubin, and altered liver function test.

Serum lactate, C-reactive protein, serum troponin, natriuretic peptide, erythrocyte sedimentation rate (ESR), and procalcitonin are used to diagnose myocarditis. Anomalies such as ST elevation, PR depression, bundle branch block, QT prolongation, and bradyarrhythmia with increasing atrioventricular nodal block have been identified in the ECGs of a small number of COVID-19 patients [51].

Myocarditis diagnosis is mainly by imaging studies echocardiogram and cardiac MRI, and definite diagnosis is by myocardial biopsy. However, further research is needed on the prevalence and risk factors, including genetic susceptibility, outcome, possible pathways, reasons of gender variances, clinical manifestations, treatment options, and protracted repercussions of myocarditis post-vaccination against COVID-19 [52].

## Conclusions

Current data indicate that younger individuals without comorbidities are more likely to experience myocarditis following SARS-CoV-2 (COVID-19) vaccinations. The vast majority of patients report chest pain, fever, and myalgia as early symptoms. Troponin, CRP, and WBC levels are all high in patients. The death rate is substantially lower than that of other causes of myocarditis and fewer than that of COVID-19 infection itself. Although infrequent, doctors should be cautious of the likelihood of myocarditis in patients who have experienced chest pain with other evitable symptoms within a few days of immunization, especially in the younger population.
